# Excessive Availability of Water for Irrigation Results in Cactus Cladode Powder with Fewer Organic Biomolecules and Reduces the Quality of the Biopolymeric Film

**DOI:** 10.1021/acsomega.4c10894

**Published:** 2025-08-04

**Authors:** Lúcio José Vieira Silva, Aline Lima Soares, Jucivânia Cordeiro Pinheiro, Brenna Karelly Almeida Lopes, Natanael Lucena Ferreira, Lucas Vinícius Pierre de Andrada, Jheizon Feitoza do Nascimento Souza, Fred Augusto Louredo de Brito, Luana Gomes Cordeiro de Araújo, José Francielson Queiroz Pereira, Andréa Monteiro Santana Silva Brito, Thieres George Freire da Silva, Ivo Diego de Lima Silva, Gloria Maria Vinhas, Adriano do Nascimento Simões

**Affiliations:** † 74384 Federal Rural University of the Semi-Arid, Mossoró 59625-900, Rio Grande do Norte, Brazil; ‡ Academic Unit of Serra Talhada, 67744 Federal Rural University of Pernambuco, Serra Talhada 56903-465, Pernambuco, Brazil; § São Paulo State Universal “Júlio de Mesquita Filho”, São Paulo 05508-220, Brazil

## Abstract

The effect of different irrigation regimes on the physical and chemical properties of cactus cladode powder and its biopolymeric films was investigated. Plants of the genera Nopalea and Opuntia were grown under four irrigation regimes of: 0, 40, 80, and 120% evapotranspiration. The cladodes were harvested, their powder was obtained, hydrated, mixed with ethanol (70%), calcium lactate (5% w/v), and dried in an oven at 55 °C for 27 h to obtain biopolymeric films. The physicochemical and spectral data related to the multivariate analysis showed that the lowest irrigation regime (0–80% of ETc) for both genders produced more acidic material, with higher levels of total phenolic compounds, total carbohydrates, and electrical conductivity. Furthermore, the surfaces of Opuntia films showed more homogeneous micro- and macrostructure and better mechanical and thermal properties compared to Nopalea. Therefore, cactus powder and films are suitable for the food and packaging production industries.

## Graphical abstract

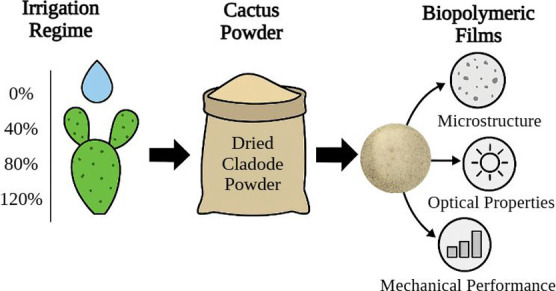

## 1 Introduction

Most synthetic plastics derived from petrochemicals resist degradation after disposal, which directly conflicts with sustainable practices. Given the increasing environmental concerns, it is crucial to explore sustainable packaging solutions. The development of eco-friendly materials utilizing biodegradable polymers is garnering significant interest, particularly for packaging applications.^1^ Furthermore, the production of biopolymers from agricultural byproducts and residues represents a global trend and one of the primary challenges of the new millennium.^2^

A biopolymer that has been extensively studied is cactus mucilage, a hydrocolloid extracted from various species of the cactus family. This biopolymer is significant due to its functional properties and its potential to extend shelf life and preserve food quality.^3^ Mucilages, also known as natural hydrocolloids, are complex polysaccharides characterized by their elastic flow. They have the ability to form molecular networks that act as barriers to oxygen and oil, exhibit water solubility, display gelling behavior, and possess emulsifying properties, making them suitable for a range of industrial applications.^2,4^ Consequently, they represent promising materials for the development of films for biodegradable packaging.^1^

Prickly pear cactus (Opuntia and Nopalea spp.) is a plant of significant importance in the semiarid regions of Brazil’s Northeast due to its widespread availability and remarkable resilience to climate conditions. It can withstand high levels of sunlight, poor soil quality, and low rainfall,^4^ making it particularly valuable as animal feed. However, the concentrations and characteristics of cactus mucilage vary depending on the genotype,^5^ environmental conditions, and harvest time.^6,7^ These variations lead to differences in the yield, viscosity, and physicochemical stability of mucilage derived from different prickly pear cactus.

Cactus mucilage is a viscous hydrocolloid primarily composed of polysaccharides found in the parenchymal tissue of cladodes. It plays a crucial physiological role in water retention and contains complex carbohydrates, proteins, and minerals.^46^ When cladodes are processed into powder, the resulting material retains the composition of the original tissue, potentially including not only mucilage but also structural components and secondary metabolites, depending on the processing and extraction conditions.^47^ Furthermore, the powder extracted from cacti may contain only mucilage when isolated using water and/or ethanol, or a mixture of constituents that complicates the standardization of formulations with a stable polymer chain. This complexity limits the application of mucilage in the development of autonomous packaging materials or in the incorporation of adjuvants that enhance the mechanical strength of the polymer matrix.^8^

Mucilage production can be influenced by seasonal variations.^9^ According to the classification of the water regime in the Brazilian semiarid region conducted by,^10^ the rainy season experiences an average monthly rainfall of 323 mm, while the dry season sees only 1 mm of rainfall. This disparity significantly affects the physicochemical properties of mucilage during these distinct periods. Pinheiro et al.^11^ observed that the rainy season and the rainy-dry transition produced biopolymeric films of superior quality. In contrast, the dry season resulted in mucilage with a higher carbohydrate content. Additionally, the availability of water during the rainy and rainy-dry transition seasons contributed to the formulation of films with enhanced mechanical properties, improved moisture barriers, a compact microstructure, and increased thermal stability. These findings underscore the substantial impact of water availability on the chemical parameters of mucilage and the structural characteristics of the resulting biopolymeric films. Therefore, the hydration of cladodes can be regarded as a consequence of irrigation, an environmental factor closely linked to the quality of biopolymeric films. Consequently, water availability during cactus cultivation affects the physicochemical characteristics of mucilage, as well as the properties of the fibers obtained during the processing of cactus powder, which in turn influences the technological properties of the biopolymeric film. We hypothesize that there may be an optimal level of irrigation in the Brazilian semiarid region that could enhance the technological properties for the subsequent production of biopolymeric films. Furthermore, the impact of water availability may vary according to each cactus genotype.

Therefore, the present study aimed to identify a suitable irrigation regime for the production of cladodes from forage cacti of the genera *Nopalea* and *Opuntia*. The objective was to obtain cactus powder for industrial applications, such as in biopolymeric films.

## 2 Materials and Methods

### 2.1 Location of the Study Area and Meteorological Conditions

The study was carried out at the Federal Rural University of Pernambuco/Academic Unit of Serra Talhada–UFRPE/UAST, at the “International Reference Center for Agrometeorology Studies of Palma and other Forage Plants”, municipality of Serra Talhada, Pernambuco, Brazil (Latitude 7°56 “20” South; Longitude 142 38°17′31” West and Altitude 499 m). The local soil is classified as Haplic Cambisol and Typical Eutrophic, according to the Brazilian Soil Classification System.^12^ The region’s climate, according to Koppen’s classification, is classified as BSwh’ Alvares et al.,^13^ with average rainfall of 642 mm year ^–1^ and atmospheric demand above 1800 mm year^–1^, with average annual values temperature and relative humidity of 24.8 °C and 63%, respectively.^14^ (Figure 1b)

**Figure 1:**
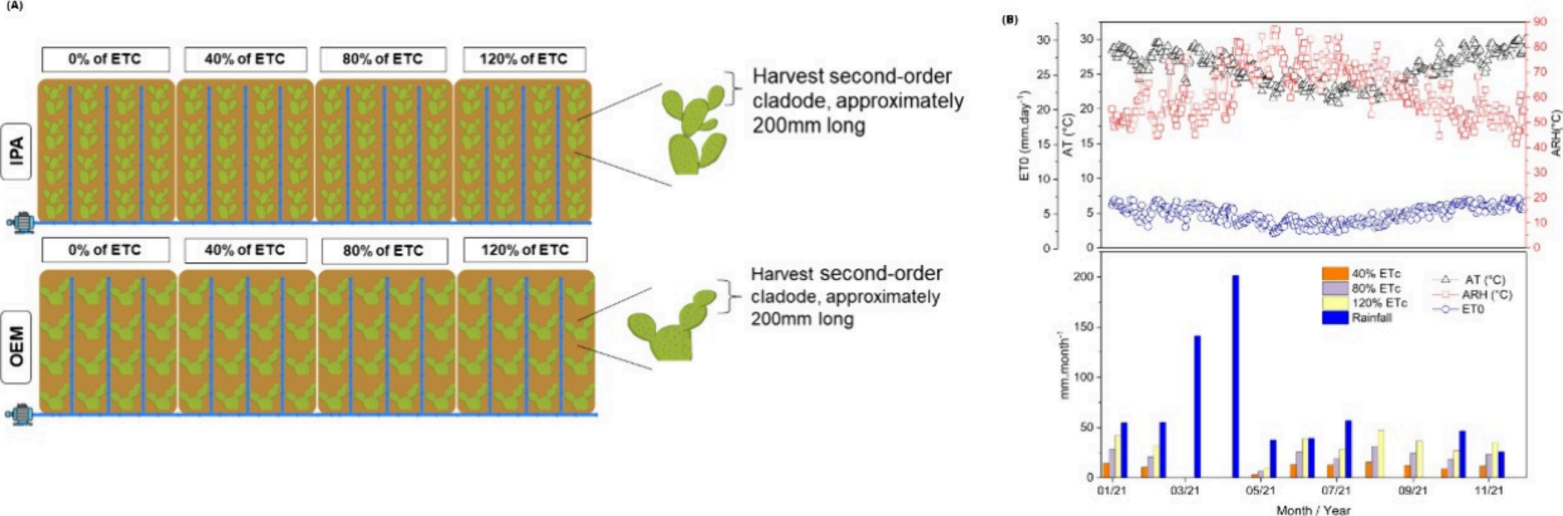
(A) Illustration of the experimental area during the cultivation of *Nopalea cochenillifera* (L.) Salm-Dyck (IPA) and *Opuntia stricta* (Haw.) Haw (OEM) under different irrigation regimes. The clones (OEM = Mexican elephant ear, and IPA) represent the subplots. (B) Meteorological conditions and water availability via irrigation during the experimental period). Note: AT: Average temperature; ARH: Average Relative Humidity.

### 2.2 Cultivation of Cactus Clones under Different Water Regimes

In this experiment, a cycle of forage cactus was analyzed, composed of two genotypes, IPA (*Nopalea cochenillifera* (L.) Salm-Dyck) and OEM (Mexican elephant ear (*Opuntia stricta* Haw.)). These were subjected to four water regimes based on crop evapotranspiration: ETc rainfed (0%); moderate water deficit (40%); light water deficit (80%); water regime higher than the water requirement of the crop (120%).

### 2.3 Field Management of Plants

When the experimental area was established, plowing, harrowing, and furrowing were conducted, along with fertilization, irrigation, and weed control throughout the experimental period. The forage palm was planted in a specific alignment, with the cladodes arranged parallel to one another and the rows positioned perpendicularly, burying 50% of the total length of the cladodes. Palm planting followed a spacing of 1.00 m between rows and 0.20 m between plants, representing a density of 50,000 plants per ha^–1^. Fertilization was maintained at a standard level, with management implemented at the beginning of each experimental cycle. The irrigation regime to be applied was based on the evapotranspiration of the main crop (i.e., forage palm), whose Kc was 0.52, by the method of Queiroz et al.^15^ To determine the reference evapotranspiration (ETc) Penman-Monteith method was used, parametrized according to FAO Bulletin 56.^16^ The meteorological data (maximum, minimum and average air temperature (°C day^–1^); average, maximum and minimum relative humidity (% day^–1^); global solar radiation (MJ m^–2^ day^–1^); wind speed (m s^–1^ day^–1^) and rainfall (mm day^–1^) were obtained from an automated weather station located ∼20 m from the experimental area, belonging to the National Meteorological Institute (INMET).

The water used in the experiment was sourced from an artesian well located approximately 50 m from the experimental areas. It was classified as C3S1, indicating high salinity.^17^ The water had an average electrical conductivity of 1.62 dS m^–1^ and a pH of 6.84, with average sodium and potassium concentrations of 168.66 and 28.17 mg L^–1^, respectively. Pest and weed control measures were implemented as needed. Irrigation was conducted using a drip system with emitters spaced 0.20 m apart, delivering a flow rate of 2.25 L h^–1^ at a pressure of 1 atm. Irrigation occurred three times a week, specifically on Monday, Wednesday, and Friday mornings.

### 2.4 Sample Preparation

Two cactus genotypes were considered: IPA (*Nopalea cochenillifera* (L.) Salm-Dyck) and Mexican elephant ear (*Opuntia stricta* (Haw.) Haw) (OEM). The average dimensions of cladodes were as follows: for the OEM genotype, 18.4 cm wide, 1.2 cm thick, and 31.6 cm long; for the IPA genotype, 11.4 cm wide, 1.49 cm thick, and 26 cm long.

They were preselected from the field, all third-order cladodes (the third generation of cladodes above the basal stem), and transported to the laboratory. There, the cladodes were washed in running water, weighed, and their lateral edges were removed. Following this, the cladodes were peeled. The material was stored in a Kraft paper bag measuring 26 cm wide × 33 cm long × 17.5 cm high and placed in an oven at a temperature of 55 °C for 15 days. After this period, the material was removed from the oven and crushed in a micro mill (Wiley R-TE-648), resulting in a brownish powder. For the ground dried cladodes analyses, the obtained powder was hydrated to a concentration of 8% w/v.

### 2.5 Physicochemical Characterization of Cactus Cladode Powder

#### 2.5.1 Powder Yield

Powder yield was quantified on a whole cladode basis for powdered obtained from fresh cladodes using eq 1:

$$\mathrm{PY}=\frac{\mathrm{Mf}(g)}{\mathrm{Mi}(g)}\times 100$$ 1

Where: PY = powder yield (%); Mi = initial mass of cladodes (g) and Mf = mass of cladode powder (g).

#### 2.5.2 Total Titratable Acidity, Hydrogen Potential and Electrical Conductivity

Total titratable acidity (TTA) was determined according to Astello-García et al.,^18^ with some modifications. The powder sample (0.8 g) was hydrated in 50 mL of water and titrated with a 0.1 N aqueous hydroxide solution (NaOH). The results were calculated with eq 2:

$$\mathrm{TTA}=\frac{N\times V\times \mathrm{Eq}\;\mathrm{malic}\;\mathrm{acid}}{M}$$ 2

Where: *N* is the normal concentration of NaOH; *V*, the volume of NaOH used for titration (mL); Eq, the equivalent in milligrams of malic acid (0.067); and *M*, the weight of the sample (g). The results were expressed as % malic acid.

The hydrogen potential (pH) was determined using a pH meter (TECNAL, TEC-5, Piracicaba, Brazil), via direct immersion of the electrode in the hydrated powder.

Electrical conductivity (EC) was measured using a conductivity meter (TECNAL, Tec-4MP, Piracicaba, Brazil). The sensor was inserted directly into the hydrated powder samples to make the reading. The results were expressed in mS cm^–1^.

#### 2.5.3 Sodium Content, Potassium Content and Phosphorus Content

The material from the Nopalea and Opuntia genotypes, after drying, was crushed in a Willey-type steel mill. and stored in labeled plastic bags. The samples were subjected to nutritional analyzes for potassium (K^+^), sodium (Na^+^) and phosphorus (P) by sulfur digestion, according to the methodology proposed by The Brazilian Agricultural Research Corporation (Embrapa).^19^

#### 2.5.4 Total Soluble Carbohydrates and Total Phenolic Compounds

The soluble carbohydrate content (TSC) was obtained according to the methodology described by ref 20 The hydrated powder (2 mL) was centrifuged (Hettich, MIKRO 220, Berlin, Germany) at 10,000 rpm, at 4 °C, for 21 min. A 10 μL aliquot of the crude sample extract was added to 490 μL of deionized water, 500 μL of 5% phenol, and 2.5 mL of 98.08% concentrated sulfuric acid, put into test tubes and shaken. Subsequently, the tubes were left to rest for 10 min in a tray containing water at room temperature. After this time, readings were taken on a spectrophotometer (Model libra S8, Biochrom, Cambridge, England) at 490 nm. The blank consisted of 500 μL of deionized water, 500 μL of 5% phenol and 2.5 mL of 98.08% concentrated sulfuric acid. The results were expressed in g of soluble carbohydrates 100 g^–1^ of dry matter and quantified based on the equation obtained for the standard curve, whose reference carbohydrate was glucose.

The determination of total phenolic content (TPC) content was carried out according to Singleton et al.,^21^ with some modifications. A volume of 2 mL of the hydrated powder was centrifuged (Hettich, MIKRO 220, Berlin, Germany) at 10,000 rpm, at 4 °C, for 21 min. A 150 μL aliquot of the supernatant was combined with 100 μL of deionized water and 250 μL of Folin Ciocalteu reagent (1N). The mixture was homogenized in a vortex (TECNAL, AP56, Araraquara, Brazil) and remained at rest for 2 min. Then, 500 μL of 20% (w/v) sodium carbonate was added, and the mixture remained at rest for another 10 min. Finally, readings were taken with a spectrophotometer (Biochrom, Libra S8, Cambridge, England) at 757 nm. To construct the analytical curve, a standard solution of gallic acid was used, in concentrations of 10, 20, 30, 40, and 50 μg mL^–1^. Total polyphenol content was expressed in micrograms of gallic acid per milligram of powder.

### 2.6 Formulation of Biopolymeric Films

In the process of developing biopolymeric films, modifications were made to the methodology proposed by^2^ to suit local conditions. The formulation process began with powder, which was hydrated at a concentration of 8% w/v, along with 40% glycerol and 5% calcium lactate to create an emulsion. The mixture was placed on a magnetic stirrer and heated to 41 °C for 10 min. Using a disposable syringe, a 35 mL aliquot of the homogenate was transferred to a plastic Petri dish measuring 9 cm in diameter and 1.5 cm in depth. Subsequently, the dish was placed in an oven for 27 h at 55 °C.

#### 2.6.1 Formulation of Plant Seedling Bags

The formulation process began with the hydration of the cladode powder, using 64 g of the powder in a mixture composed of 400 mL of distilled water and 400 mL of 70% (v/v) ethanol, forming an emulsion. This mixture was then heated to 41 °C on a magnetic stirrer for 15 min. After this period, 40% glycerol and 5% calcium lactate were added, followed by continuous stirring for an additional 15 min to ensure complete homogenization. Subsequently, 750 mL of the homogenized solution was transferred into a plastic tray measuring 20 cm in width and 33 cm in length. The tray was then placed in an oven at 55 °C for 48 h to form the seedling bags.

### 2.7 Physicochemical Characterization of Biopolymeric Films

#### 2.7.1 Fourier Transform Infrared Spectroscopy (FTIR)

Spectral analyses in the mid-infrared region were carried out in a Fourier transform infrared (FTIR) spectrophotometer (PerkinElmer Frontier model), using the universal attenuated total reflection (UATR) accessory. The spectra were acquired in the region of 4000–400 cm^–1^, resolution 4 cm^–1^ and 8 scans. The blank was air and the measurements were taken directly on the biofilm, under the diamond crystal. The spectra were also analyzed using the principal component analysis (PCA) multivariate exploratory analysis method, which provides information about the similarities and differences found in the data set.

#### 2.7.2 Water Vapor Permeability

WVP was measured according to ref 2 with some modifications. The film samples were placed in sealed cells of permeable membrane permeation cells (BACKER 50 mL) containing around 15 g of calcium carbonate. The well-sealed systems were placed in a desiccator containing saturated sodium chloride solution (75% relative humidity) at a temperature of 25 °C. The systems were weighed over a period of 7 days at fixed time intervals. PVA was expressed in (g mm m^–2^ d kPa) calculated using eq 3

$$\mathrm{PVA}=\frac{\mathrm{WVT}R\times X}{\Delta p}$$ 3

where: WVTR (g m^–2^ d) is the water vapor transmission rate defined as the change in weight as a function of time (g d^–1^) (calculated by linear regression (*R* ^2^ > 0.99)) divided by the transfer area (m^2^); *X* (mm) is the thickness of the film; Δ*p* (kPa) is the difference in the partial pressure of water vapor across the film (Δ*p* = *p* (RH2 – RH1) = 2.38 kPa, where *p* is the saturation vapor pressure of water at 25 °C, RH 2 = 75% and RH 1 = 0%).

#### 2.7.3 Water Solubility and Moisture Content

Water solubility was measured using 2.0 cm × 2.0 cm biofilm fragments, dried in an oven at 55 °C for 24 h, cooled to room temperature in a desiccator, weighed and immersed in 50 mL of distilled water at 25 °C for 30 min. After that, the undissolved fragments were stored in the oven for 24 h at 55 °C, placed in the desiccator to cool and weighed at the end of the process. Water solubility was determined by eq 4:

$$\mathrm{SW}=\frac{\mathrm{Mi}-\mathrm{Mf}}{\mathrm{Mi}}\times 100$$ 4

Where: SW = solubility in Water (%); Mi: Initial mass of the fragments (g); Mf*:* Final mass of the fragments (g).

Moisture content was measured by cutting the films into 2.0 cm × 2.0 cm squares and weighing them. After that, they were taken to the oven for 24 h at 55 °C until constant weight (dry sample weight). The final weighing of the fragments determined the moisture content of the biofilms, calculated by eq 5:

$$\mathrm{MC}=\frac{\mathrm{Mi}-\mathrm{Mf}}{\mathrm{Mi}}\times 100$$ 5

Where: MC: Moisture Content (%); Mi: Initial mass of the fragments (g); Mf: Final mass of the fragments (g).

### 2.8 Optical and Microstructural Characterization of Polymeric Films

#### 2.8.1 Color

The color was obtained using a calorimeter (RS - 232 with serial output RGB - 1002) with values from the RGB system. The data obtained by the calorimeter were divided by 4 to adapt to the RGB scale (0–255) and then converted to the CIE L*, a*, b* color scale.^22^ Where L* corresponds to variations in the luminosity of the sample (0–100, darkest to brightest), a* corresponds to variations from green (−*a*) to red (+*a*), and b* is attributed to variations between blue (−*b*) in yellow (+*b*). Value conversion was performed using online software available on a public Web site [http://www.easyrgb.com/en/convert.php#Result]. Subsequently, the a* and b* data set were converted and expressed as Chroma saturation values (C*) according to the methodology of Espino-Díaz et al.,^23^ in which:

$$C^{*}=(a^{*2}+b^{*2})1/2$$ 6

#### 2.8.2 Scanning Electron Microscopy

The surface morphology of the films was observed by scanning electron microscopy (SEM) using a scanning electron microscope (3400N SEM, S) under standard high vacuum conditions at a voltage of 5 kV. Film samples were sprayed with gold particles for image contrast.

### 2.9 Mechanical Properties and Film Thickness

#### 2.9.1 Tensile Strength

Tensile strength (TS) was performed using a tensile machine (IMPAC, IP-AEL-A-50, São Paulo, Brazil) according to the method proposed by ref 2, with modifications. For each film formulation, 3 rectangular strips of film (20 mm × 70 mm) were tested at a head speed of 100 mm/min using a double clamp with a separation of 50 mm.

#### 2.9.2 Thickness

The thickness (in mm) was measured at 10 random points on the films with a digital micrometer, with a resolution of 1 μm, and an average was taken.^2^

### 2.10 Thermal Properties

The thermal stability of the films was evaluated using the TGA2 thermal analysis system (Mettler Toledo). The experiment was carried out under a nitrogen atmosphere, with a heating range from 35 to 600 °C, at a heating rate of 10 °C min^–1^ for each sample.

### 2.11 Statistical Design and Analysis

The experimental design used was randomized blocks (DBC), in a 4 × 2 factorial scheme (in split plots), and four replications. Each plot was characterized by a water regime, while the subplots were made up of cactus clones. Each subplot was composed of four rows with 25 plants each, arranged in an area of 20 m^2^. The plots were composed of 2 subplots, totaling an area of 40 m^2^.

The first experimental cycle began in January 2017, lasting until mid-June 2018. The second cycle began in February 2019 and ended in August 2020. Both lasted approximately 18 months. Two more experimental cycles were conducted, with the standardization cut being carried out in November 2020 and the harvest in January 2022.

The analyses were carried out in quadruplicate with the data subjected to normality tests and Tukey’s test at 5% probability, with the aid of R software, version 4.2.1. For the regression analyses, Sigma Plot software version 14 was used; the models were chosen based on the significance of the regression coefficients, adopting the 1% probability level and the determination coefficients (*R* ^2^). The graphs were created using Sigma Plot version 14 and OriginLab version 8.5 software. For principal component analysis (PCA), the software tool R (R CORE TEAM, 2022) was used, in which the data means of the studied properties were decomposed into sets of orthogonal vectors. Matlab (MATLAB R2010a 7.10.0.499, MathWorks) and the PLS Toolbox package (eigenvector Research, Inc.) were also used. The results of the correlation matrix were displayed in biplots, with their distribution in the space of ordinations, variances and Pearson correlation.

## 3 Results and Discussion

### 3.1 Irrigation Regime Modifies the Organic and Mineral Composition of the Cladode Powder of OEM and IPA

The analysis of powder yield indicated that the OEM genotype produced a higher amount of dried cladode powder than the IPA genotype (Table 1). When comparing irrigation regime, there was no significant interaction (Table 1).

**Table 1:** Powder Yield of Cladodes from *Nopalea cochenillifera* (L.) Salm-Dyck (IPA) and *Opuntia stricta* (Haw.) Haw (OEM) Cultivated under Different Irrigation Regimes (Simple Effect)^a^

**interaction effect**		
**regime**	**cladode powder yield %**	
**(% of ETc)**	**IPA**	**OEM**
0	1.508 ± 0.696 Ba	1.912 ± 1.11 Aa
40	0.998 ± 0.228 Ba	2.792 ± 1.013 Aa
80	1.228 ± 0.283 Ba	1.484 ± 0.101 Aa
120	1.311 ± 0.540 Ba	1.512 ± 0.360 Aa

a Note: Different letters indicate statistical difference between the means using the Tukey test (*p* < 0.05), capital letters between clones, lower case letters between irrigation regime.

Cactus harvesting yields low powder content, complicating its industrial application. This study produced results consistent with previous findings in the literature regarding the cactus genus Nopalea. In contrast, the genus Opuntia demonstrated higher powder yields. Variations in cactus powder performance may be attributed to several factors, including the extraction method, the age and size of the cladodes, climatic conditions, and the management practices employed, all of which can influence the quantity and quality of the material obtained.^3,4^

The IPA genotype exhibited lower TTA values; conversely, it demonstrated higher pH and electrical conductivity (EC) (Figure 2A–C). Additionally, there was a tendency for OEM to show increased acidity and EC as the irrigation regime rose to 80% of the crop evapotranspiration (ETc) (Figure 2A,C).

**Figure 2:**
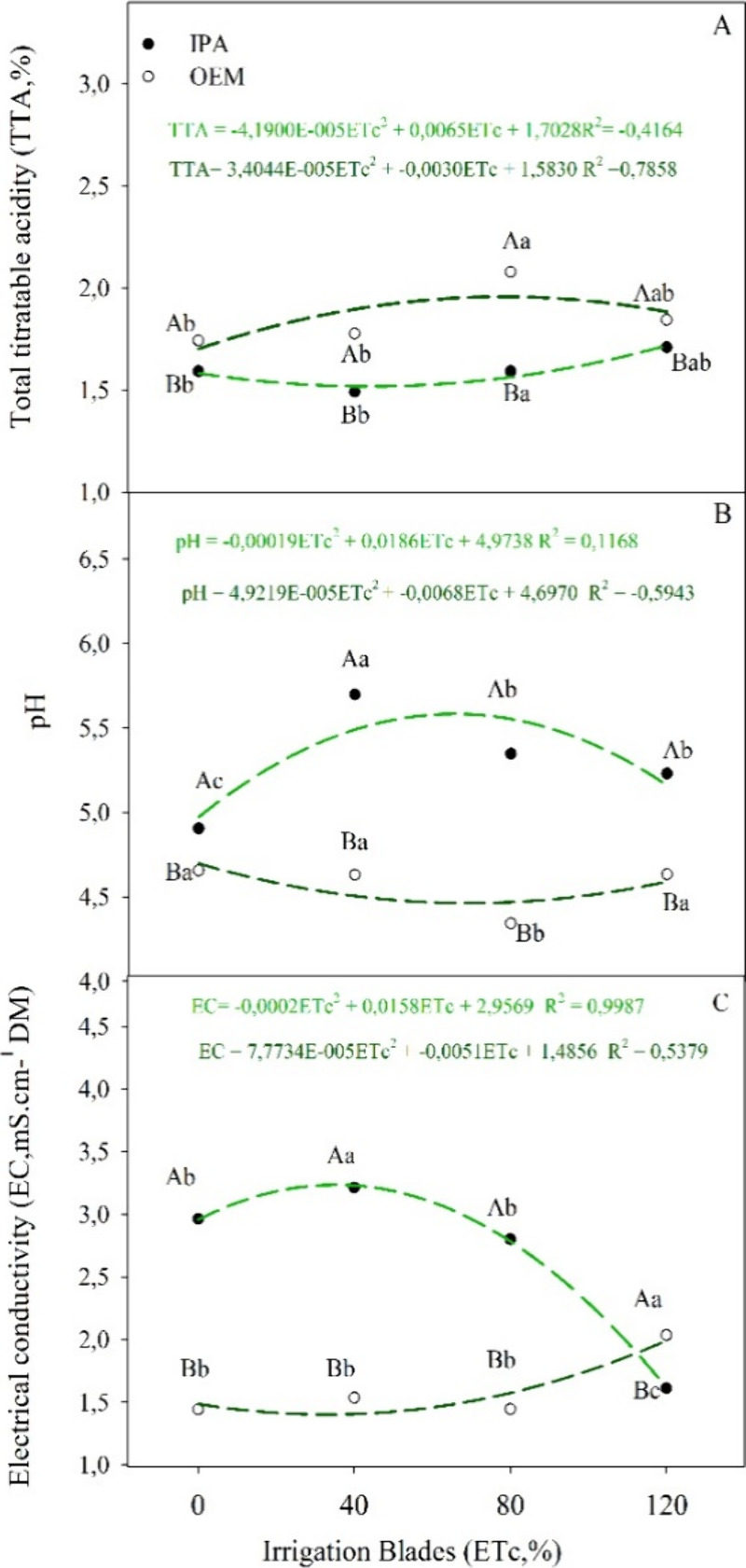
(A) Total titratable acidity, (B) pH, and (C) electrical conductivity of cladodes from *Nopalea cochenillifera* (L.) Salm-Dyck (IPA) and *Opuntia stricta* (Haw.) Haw (OEM) cultivated under different irrigation regimes. Note: different letters indicate a statistical difference between the means by Tukey’s test (*p* < 0.05), uppercase letters between clones, lowercase letters between irrigation regime.

The examination of the results from TTA indicated a correlation between pH and EC. As the irrigation depths increased, both pH and EC decreased proportionally, which contributed to the proportional increase in TTA. The pH range observed in the current study was slightly acidic, measuring between 4.47 and 5.7.^23^ investigated the variation of pH in cactus-based mucilage and found that films produced with a pH between 5.0 and 7.0 exhibited superior mechanical properties and thickness. It is important to note that ref 23. Obtained purified mucilage through soaking in water and precipitation with ethanol, while in this study, the powder was obtained directly from dried cladodes without mucilage extraction. Electrical conductivity is utilized to estimate the concentration of ions, a factor that directly influences the viscosity of the mucilage, thereby affecting its processing and its capacity to produce biopolymeric films. It is known that the addition of positive ions tends to reduce repulsion and, consequently, molecular expansion, resulting in decreased viscosity.^24^ The concentrations of ions such as K^+^, P, and Na^+^ were also quantified in the current study (Figure 3C,D, Table 2). Notably, these ions exhibited a slight increase in the irrigation regime. In addition to Na^+^, K^+^, and P, the powder also contains calcium and magnesium ions,^25^ which may account for the slight decrease in electrical conductivity.

**Figure 3:**
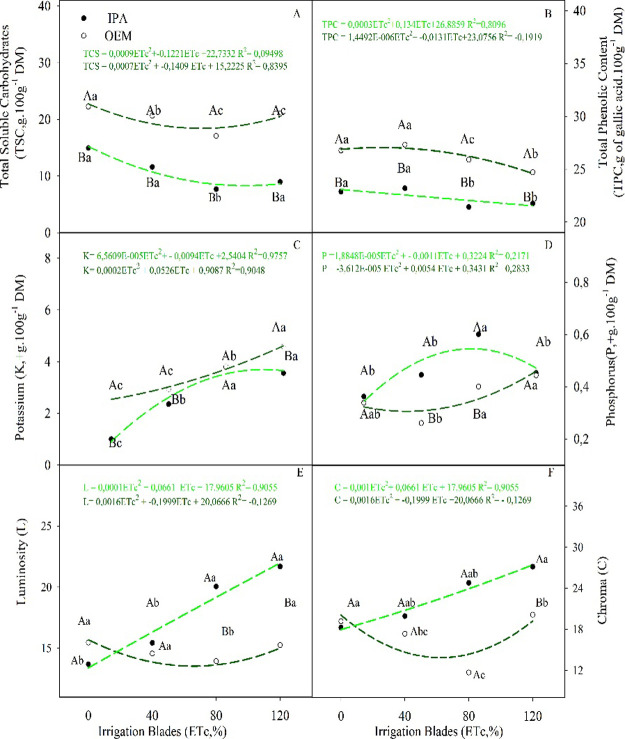
(A) Total soluble carbohydrates, (B) total phenolic content, (C) potassium content, (D) phosphorus content, (E) luminosity, and (F) color of cladodes from *Nopalea cochenillifera* (L.) Salm-Dyck (IPA) and *Opuntia stricta* (Haw.) Haw (OEM) cultivated under different irrigation regimes. Note: different letters indicate a statistical difference between the means by Tukey’s test (*p* < 0.05), uppercase letters between clones, lowercase letters between irrigation regime.

**Table 2:** –Sodium Content of Cladodes from *Nopalea cochenillifera* (L.) Salm-Dyck (IPA) and *Opuntia stricta* (Haw.) Haw (OEM) Cultivated under Different Irrigation Regimes (Interaction Effects of Irrigation Regime Versus Genotype)^a^

**interaction effect**		
**regime**	**Na** ^**+**^	
**(% of ETc)**	**IPA**	**OEM**
0	0.098 ± 0.00 Ba	0.150 ± 0.01 Aa
40	0.101 ± 0.01 Aa	0.101 ± 0.00 Ab
80	0.116 ± 0.01 Aa	0.107 ± 0.02 Ab
120	0.109 ± 0.00Aa	0.096 ± 0.00 Ab

a Note: Different letters indicate statistical difference between the means using the Tukey test (*p* < 0.05), capital letters between clones, lower case letters between irrigation regime.

The IPA genotype generally exhibited lower levels of TSC, TPC, and K^+^ compared to the OEM genotype (Figure 3A–C). In contrast, the IPA genotype demonstrated higher phosphorus levels at ETc rates of 40 and 80% (Figure 3D). For Na^+^, only the OEM genotype in the control group showed a significant difference compared to the IPA genotype (Table 2). Additionally, it is evident that the gradual increase in irrigation regime, as indicated by the rise in ETc, resulted in a corresponding decrease in TSC and TPC levels for both clones (Figure 3A,B). Conversely, potassium and phosphorus levels increased gradually (Figure 3C,D). This trend for Na^+^ was only observed between the control irrigation regime and the other treatments in the OEM genotype (Table 2).

The higher phenolic content in the powder of OEM compared to IPA has been previously documented.^6,7^ The reduction in TPC at higher irrigation levels may be linked to decreased stress, as the accumulation of secondary metabolites is influenced by both biotic and abiotic factors.^18^ However, from the perspective of producing mucilage for incorporation into food products, a biopolymer with elevated levels of phenolic compounds is desirable, as it enhances the antioxidant potential of the food, as reported by Nabil et al.^26^ The highest TSC values were observed in the OEM genotype. As irrigation levels increased, there was a corresponding decrease in TSC (Figure 3A). This may be attributed to increased water availability reducing the water stress on plants, which in turn affects mucilage synthesis. Under conditions of abundant water, cacti tend to produce mucilage with a lower concentration of soluble solids, such as carbohydrates and phenolic compounds, leading to variations in mucilage composition in response to rainfall patterns in the region.^27^

Minerals play a crucial role in plant development. Potassium (K^+^), phosphorus pentoxide (P_2_O_5_), and sodium (Na^+^) are essential for various physiological processes in plants, including metabolism and photosynthesis. For instance, cacti exhibit a higher absorption of K^+^ from the soil, indicating that this nutrient is vital for activating enzyme systems and facilitating photosynthesis.^28^ The literature reports that K^+^ levels in cacti range from 19.4 to 65.8 g kg^–1^ of DM. Moritani et al.^29^ demonstrate that potassium content is critical for metabolic processes and plant growth under nonsaline conditions. The present study observed a significant increase in K^+^ and P^–^ content in prickly pear cactus powder with rising irrigation regime levels. This phenomenon can be attributed to the accumulation of K^+^ in the irrigation water, which was sourced from an artesian well with a potassium concentration of 28.17 mg L^–1^. Additionally, there was a decrease in Na^+^ concentration with increasing depth (Table 2). This decline is likely due to the mineral imbalance in the composition of the cactus pear, where the Na^+^ content is lower, despite the irrigation water having a high sodium concentration of 168.66 mg L^–1^.

Based on the results of this study, increasing the irrigation regime during plant cultivation did not lead to a significant enhancement in cladode powder yield. Furthermore, carbohydrates and total phenolic compounds exhibited lower levels at higher irrigation regimes, rendering them less suitable for mucilage production intended for incorporation into food, as these components are crucial for food composition. Conversely, higher irrigation regimes resulted in an increase in essential inorganic elements such as K^+^ and P^–^, which must be considered depending on the intended use of this biopolymeric film, particularly in animal feed. Additionally, the OEM species demonstrates strong mucilage adaptation potential and is well-suited for food applications due to its higher content of carbohydrates and phenolic compounds, as well as important minerals like potassium, compared to the IPA genotype.

### 3.2 An Increase in Water Availability for Plants Reduces the Quality of Biopolymeric Films

Water vapor permeability and thickness were significantly higher in the IPA genotype compared to the OEM genotype (Figure 4A and Table 3). On the other hand, the OEM genotype produced more resistant films than IPA, regardless of the irrigation regime used (Figure 4C). Furthermore, it is noteworthy that increasing the irrigation regime, at least up to 80% of ETc, resulted in an increase in water solubility, and moisture content, while simultaneously reducing tensile strength (Figure 4).

**Figure 4:**
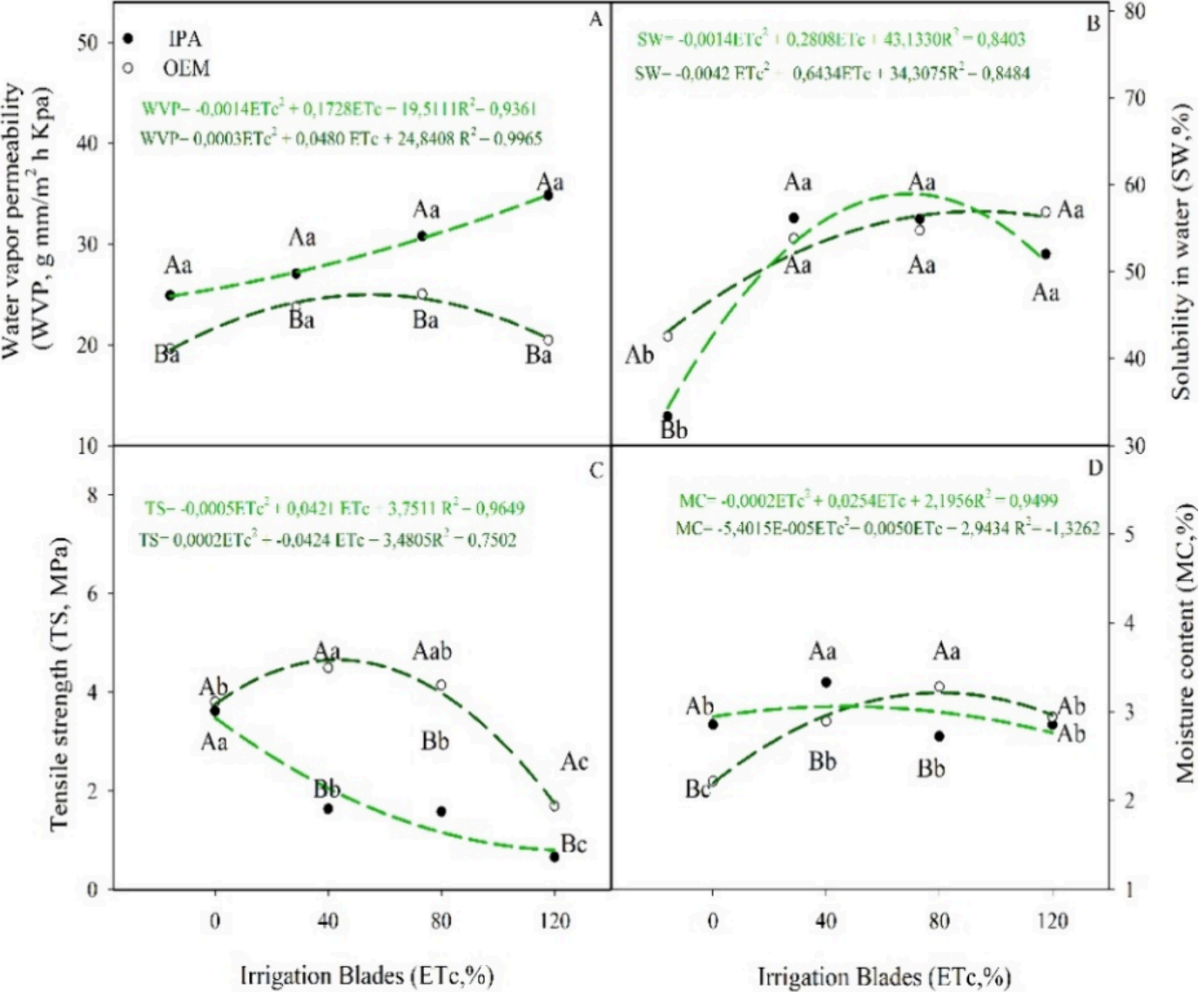
(A) Water vapor permeability, (B) water solubility, (C) tensile strength, and (D) moisture content of biopolymeric films based on cladode powder from *Nopalea cochenillifera* (L.) Salm-Dyck (IPA) and *Opuntia stricta* (Haw.) Haw (OEM), cultivated under different irrigation regimes. Note: different letters indicate a statistical difference between the means using Tukey’s test (*p* < 0.05), uppercase letters between clones, lowercase letters between irrigation regime.

**Table 3:** Thickness of Biopolymeric Films Based on Cladode Powder from *Nopalea cochenillifera* (L.) Salm-Dyck (IPA) and *Opuntia stricta* (Haw.) Haw (OEM) Cultivated under Different Irrigation Regimes (Interaction Effects of Irrigation Regime Versus Genotype)^a^

**interaction effect**		
**regime**	**thickness**	
**(% of ETc)**	**IPA**	**OEM**
0	0.611 ± 0.06 Ab	0.663 ± 0.01 Aa
40	0.646 ± 0.01 Ab	0.582 ± 0.01 Ba
80	0.742 ± 0.03 Aab	0.589 ± 0.04 Ba
120	0.809 ± 0.02 Aa	0.577 ± 0.16 Ba

a Note: Different letters indicate a statistical difference between the means using the Tukey test (*p* < 0.05), uppercase letters between clones, lowercase letters between irrigation regime.

For the application of films and coatings, it is essential for the WVP of the film to be as low as possible, as this provides protection and minimizes the transmission of moisture between the atmosphere and the food.^30^ Although no statistically significant differences were observed among irrigation regimes for water vapor permeability (WVP), a trend toward lower WVP values was noted at the lowest irrigation level. The lower WVP observed in the OEM clone compared to the IPA can be attributed to the more homogeneous microstructure achieved in the OEM polymer films (Figure 8). Furthermore, the moisture content of the polymer films was lower at the evapotranspiration control irrigation regime in both genotypes examined.

Materials used in coatings and biofilms are classified into two categories: hydrophobic and hydrophilic.^31,32^ In this study, forage palm powder, which is rich in polysaccharides, is categorized as a hydrophilic material. This type of material typically exhibits good water solubility, which is advantageous for dispersing solutes and forming more homogeneous films. Water solubility is a crucial property of polymeric films, as certain applications, such as packaging, may require reduced solubility in water to enhance product integrity and water resistance.^33^ This solubility reflects the film’s behavior when exposed to water or humid environments. The measurements in this study indicated that the two genotypes demonstrated low water solubility when the films were produced with irrigation regime at 0%.

To measure the mechanical properties of the films, tensile strength (TS) analysis was employed, as it indicates the maximum tension that a material can withstand when stretched or pulled until it breaks.^34^ According to ref 1, materials used for packaging must possess sufficient tensile resistance to maintain the integrity of the products they contain. The results clearly demonstrated the significance of limited irrigation in enhancing the properties of the films. Generally, cactus films exhibit low resistance.^2^ In this case, the 120% ETc layer reduced the resistance of biopolymeric films by more than 50% (Figure 4C) and increased permeability, which poses a challenge for cactus films due to their high solubility in water. The lower permeabilities observed with minimal irrigation may be attributed to the higher concentration of carbohydrates, resulting from their tightly packed network structures stabilized by hydrogen bonds.^35^ When comparing the TS rates found in the polymeric films from each of the genotypes studied, the OEM genotype stood out, exhibiting a significantly higher TS. This characteristic can be attributed to the more uniform surface of this film, which has fewer pores and fissures (Figure 8). Cladodes of the genus *Nopalea* were able to form thicker biopolymeric films than those of the genus *Opuntia* (Table 3). This property indicates the satisfactory durability of the polymer and its suitability for packaging.^2^ However, the effectiveness of polymer films depends on their overall properties.^36^

Thermal degradation was observed, and the stages of deterioration of biopolymeric films harvested under four irrigation levels were noted in the temperature range from 35 to 600 °C (Figure 5). The IPA 40% genotype recorded the highest final degradation temperature at Stage I, indicating that the water molecules were more effectively adhered to the polymer matrix. This stage corresponds to the volatilization of moisture.^37^ The IPA 120% exhibited less mass loss in both the first and last stages of degradation, concluding the analysis with minimal mass loss. The 0% regime of the IPA genotype recorded the highest maximum degradation temperature at the end of the fourth stage. When evaluating the effect of the OEM genotype, the lowest mass loss observed across all degradation stages was notable for the 0% irrigation level. Conversely, the 120% regime supported a higher maximum degradation temperature but experienced greater mass loss by the end of the analysis.

**Figure 5:**
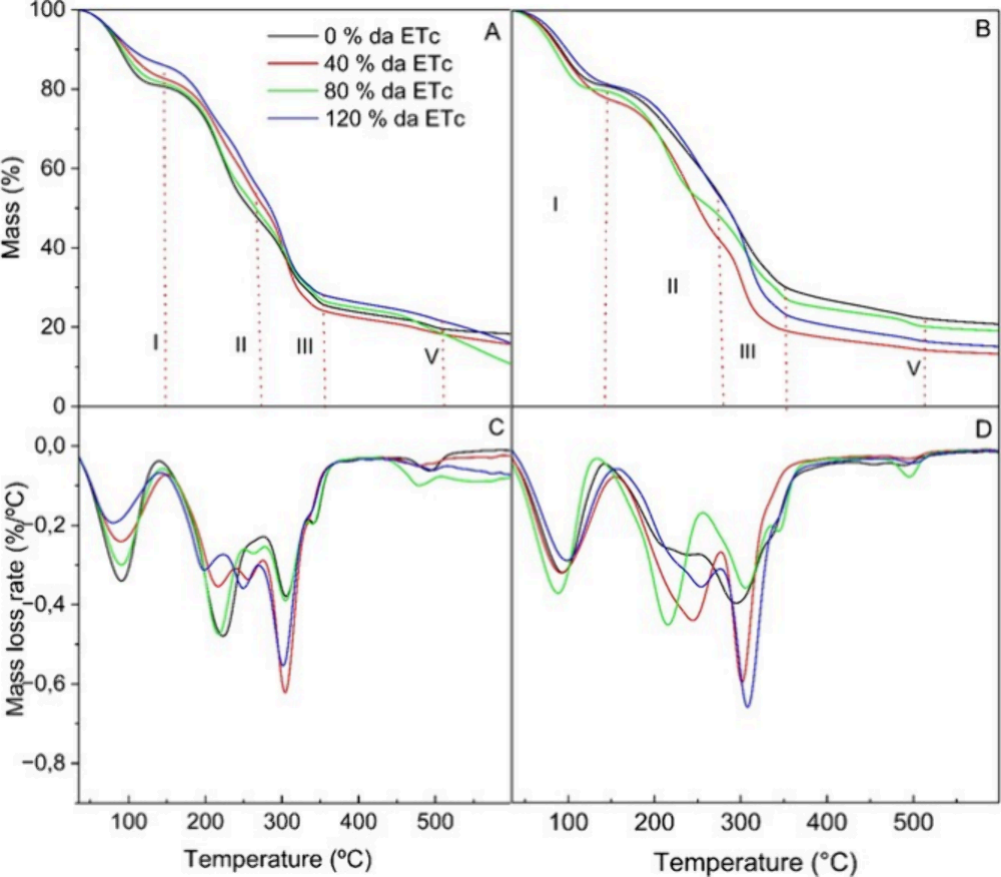
**-** Residual mass and DTG curves of films based on cactus cladode powder from *Nopalea cochenillifera* (L.) Salm-Dyck (IPA) (A, C) and *Opuntia stricta* (Haw.) Haw (OEM) (B,D), cultivated under different irrigation regimes.

Macrostructurally, the biopolymer films indicated that the IPA genotype exhibited lighter colors compared to the OEM genotype. The films became progressively lighter as the irrigation regime increased in both genotypes studied, with the exception of the irrigation regime at 80% of ETc in the OEM genotype (Figure 6). This observation is supported by the analysis of the luminosity and chroma graphs, which reveal higher values for the IPA genotype and an increase in the presence of irrigation rates. Microstructurally, all the films studied displayed spherical aggregates, indicating the agglomeration of powder particles.^38^ However, a greater density of craters was observed in the polymeric films of cladodes grown at 120% of ETc (Figure 7). This may be related to a significant decrease in the resistance of these films (Figure 4C).

**Figure 6:**
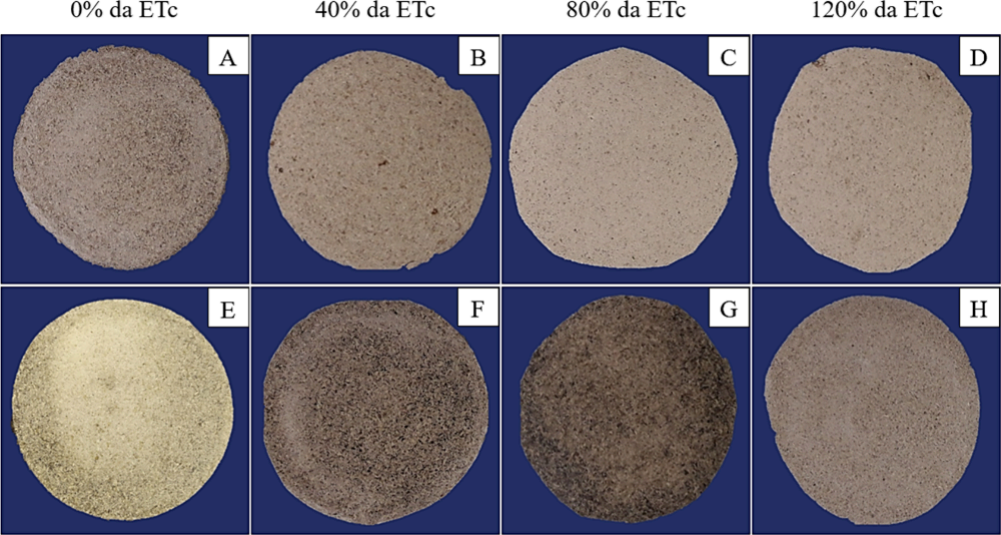
Visual appearance of biopolymeric films based on mucilage from *Nopalea cochenillifera* (L.) Salm-Dyck (IPA) (A–D) and *Opuntia stricta* (Haw.) Haw (OEM) (E–H) cultivated under different irrigation regimes.

**Figure 7:**
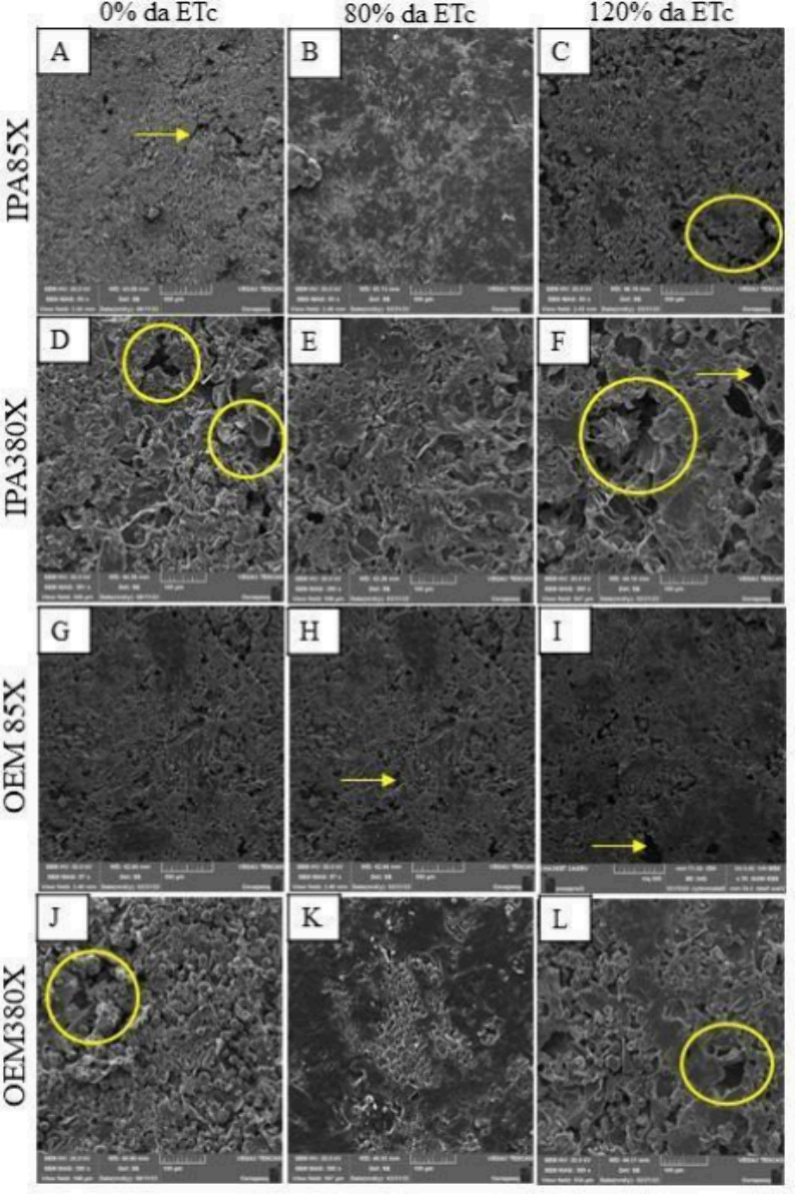
Scanning electron microscopy (SEM) images of biopolymeric films based on cladode powder from *Nopalea cochenillifera* (L.) Salm-Dyck (IPA) (A–F) and *Opuntia stricta* (Haw.) Haw (OEM) (G–L) cultivated under different irrigation regimes.

The films derived from the powder of the IPA genotype exhibited lighter tones compared to those of the OEM (Figure 6). This difference may be attributed to the pigment content in the OEM genotype relative to that of IPA,^6^ as indicated by the higher photometric values for IPA compared to OEM (Figure 6A,B), which are lower than those reported for *O. ficus-indica* (69–99 L*).^39^ The lower values observed in this study compared to the literature may be linked to the extraction method employed. Typically, studies such as those by^27,42^ describe the extraction of mucilage using a solvent, followed by drying through forced-air oven dehydration. Furthermore, scanning electron microscopy (SEM) revealed differences in the homogeneity of biopolymeric films derived from IPA, which exhibited greater uniformity than those from OEM (Figure 7). Micrographs at 85× magnification show the inhomogeneous particles of the IPA genotype biofilm. These points, fissures, or pores can facilitate gas exchange, which is significant because when mucilage is incorporated into foods, it prevents them from entering anaerobic and fermentative pathways.^38^ However, when the resolution was increased to 380x (Figure 7), it was observed that the IPA surface appeared more heterogeneous than the OEM surface, which demonstrated smoother structures with fewer agglomerates. This difference in structure can directly influence other properties of the biofilms.

The results of infrared analysis (FTIR) of IPA and OEM powder demonstrated similar behavior in both species studied, a characteristic also common to biopolymeric films. FTIR spectroscopy provided a detailed chemical characterization of the material by correlating its absorption frequencies with known binding absorption frequencies.^41^ The FTIR spectra of the powder and polymeric film (Figure 8) were comparable to those recorded in previous studies.^2,42^ The broad absorption band around 3342 cm^–1^ corresponds to the OH stretching of the alcohol and carboxylic acid −OH groups that participate in intermolecular hydrogen bonding,^2,41,43^ demonstrating the affinity of biofilms for water molecules. Absorption bands at 2932 and 2885 corresponded to asymmetric and symmetric vibrations of CH_2_ or CH_3_ bonds, respectively. According to,^44^ the elongation of these bands represents the presence of cellulose in the film formulation. The low intensity of the absorption peak at 1730, attributed to the C=O stretching vibration of the nonionized carboxyl group, correlated with the appearance of peaks 1625 and 1418, attributed to the COO^–^ asymmetric and symmetric stretching properties. Properties of carboxylic acid salts present in mucilage^41,42^ showed that the carboxyl group ionized, forming a salt.^2,40^ Reported that peaks were observed at 1370, 1321, and 1244 that correspond to the C–H or O–H vibration. The strong peak at 1030 is characteristic of polysaccharides, representing C–O–C or C–O–H vibrations.^45^

**Figure 8:**
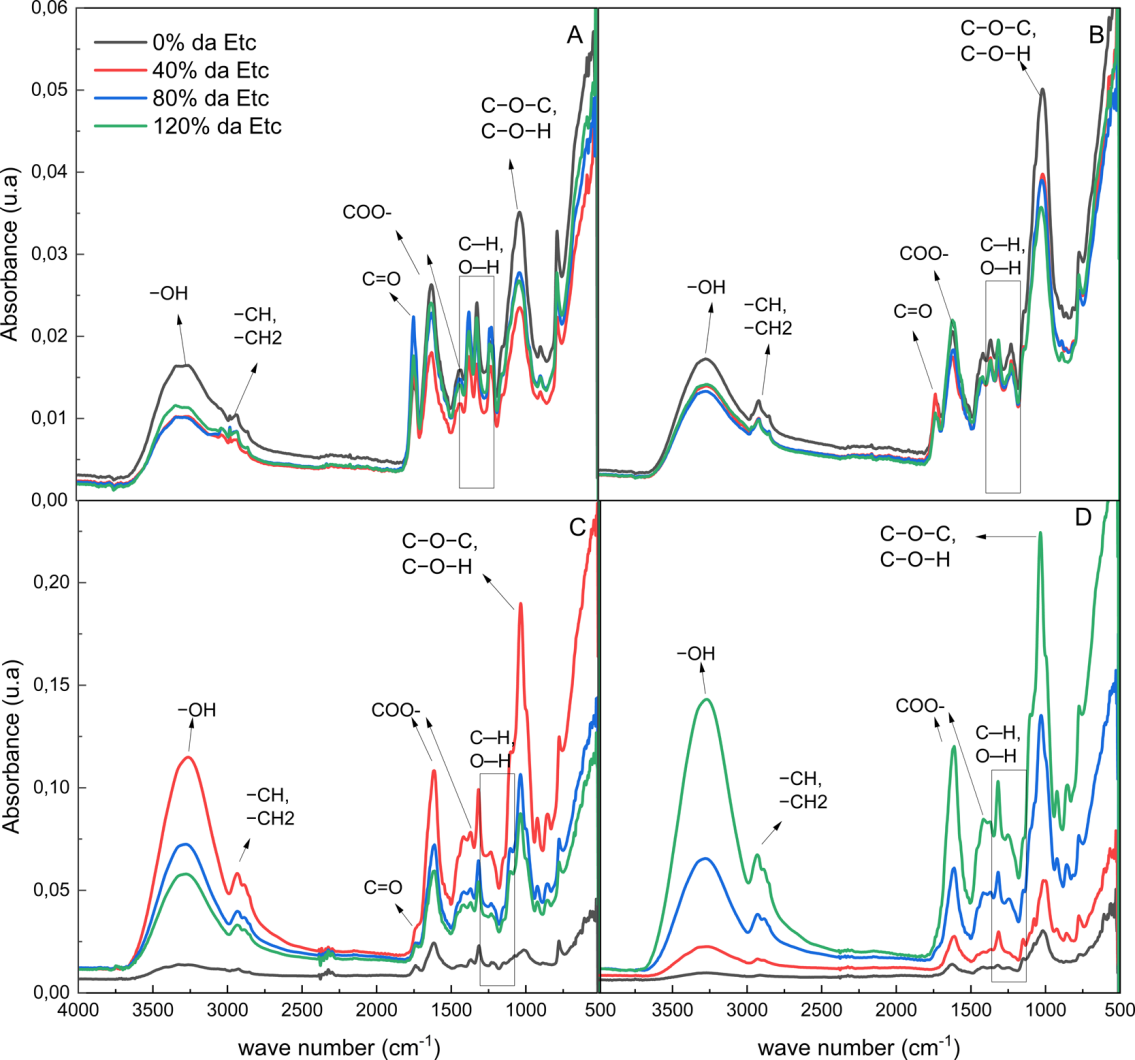
FTIR spectra of cladode powders (A, B) and biopolymeric films (C, D) based on *Nopalea cochenillifera* (L.) Salm-Dyck (IPA) (A, C) and *Opuntia stricta* (Haw.) Haw (OEM) (B, D) cultivated under different irrigation regimes.

From the direct evaluation of the spectral profiles of the films, the chemical characteristic of each type of film was determined. Next, multivariate analyses were carried out in the form of principal components (PCA) (Figure 9). Initially, PCA was performed considering discrete physicochemical data and then another PCA considering infrared data. Figure 10, presents the first two main components (PC1 and PC2) for the samples from the treatments carried out, and the physicochemical parameters evaluated in the powder and biopolymeric film. For PC1, the variation was explained with a greater contribution from the variables TSC (0.95), Na^+^ (0.93), TS (0.81), MY (0.73), P (−0.75), TH (−0.79), WVP (−0.84) and pH (−0.89). Thus, the treatments IPA (80%), IPA (120%), OEM (40%) and IPA (40%), which contributed most to the variation in PC1, can be related to these characteristics. Based on the distribution of treatments in PC1, treatments IPA (40%), IPA (80%) and IPA 120%) presented powder with higher pH, lower MY, TSC and Na^+^, while their biopolymeric films had higher WVP and lower TS. The OEM treatment (40%) which obtained higher MY, TSC and Na^+^, correlated with biopolymeric films with higher TSC and lower WVP. PC2 was characterized with greater contributions from the variables: K^+^ (0.97) and EC (0.84) and SW (0.84). According to the distribution of these variables in PC2, the OEM treatment (120%) was associated with powder with higher K^+^, EC and biopolymeric film with higher SW.

**Figure 9:**
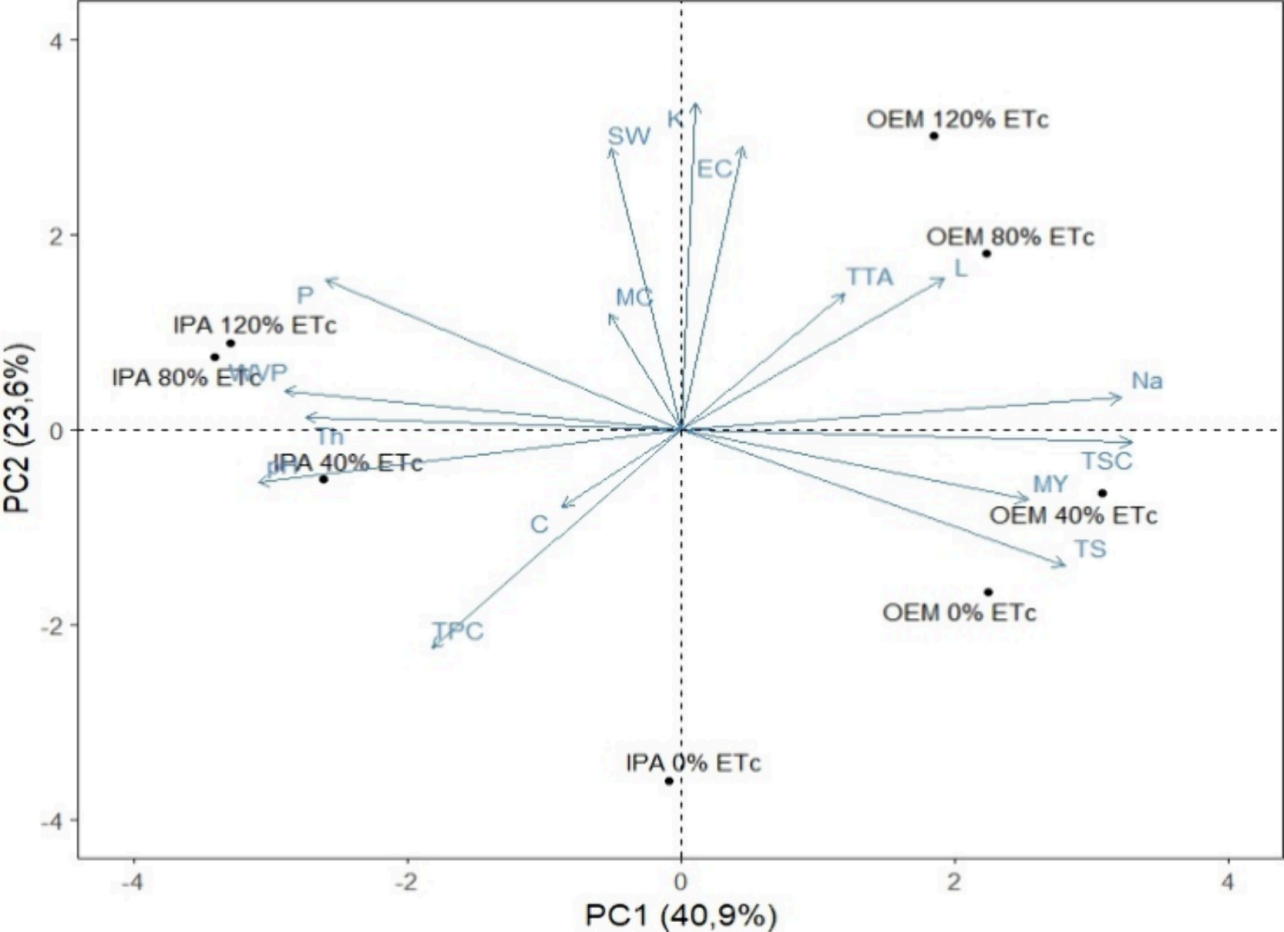
PCA biplots based on standardized means of powder and polymeric film variables, immediately after harvesting the cladode and after treatments 0% of ETc, 40% of ETc, 80% of ETc, 120% of ETc for each species studied, namely, *Nopalea cochenillifera* (L.) Salm-Dyck - IPA and *Opuntia stricta* (Haw.) Haw - OEM. Note: EC: electrical conductivity; TPC: phenolic content; FTIR: infrared spectroscopy; TTA: total titratable acidity; P: potassium; K^+^: phosphorus; Na^+^: sodium; TSC: total soluble carbohydrates; TS: tensile strength; MC: moisture content; SW: solubility in water; WVP: water vapor permeability; TH: thickness.

**Figure 10:**
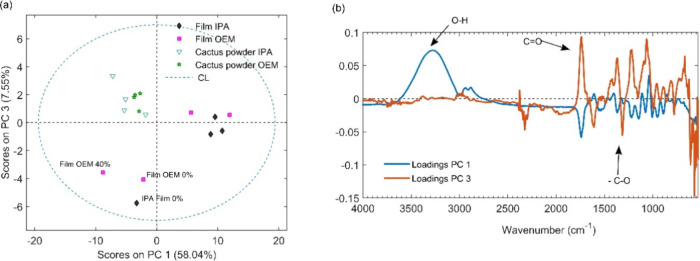
Analysis of spectral components in cladode powder and polymeric films: *Nopalea cochenillifera* (L.) Salm-Dyck–IPA and *Opuntia stricta* (Haw.) Haw–OEM. (A) Biplots of principal component analysis (PCA) showing the distribution of samples based on scores of PC1 and PC3. (B) Loadings of PC1 and PC3 indicating the main functional groups (O–H, C=O, and C–O) contributing to spectral differences among the samples.

The powder and film spectra were initially preprocessed using standard normal variate (SNV) normalization and smoothed with a Savitzky-Golay filter (with 15 points and a polynomial of order 2) to reduce spectral noise and correct for additive and multiplicative effects, such as baseline variations. The PCA score graph (Figure 10A) indicates that the powder samples differ from the films primarily along the first principal component (PC1), largely due to the hydroxyl (OH) and carbonyl (C=O) bands, as illustrated in the loadings graph in Figure 10B. The film samples exhibit a positive correlation with the hydroxyl band in PC1, while the powder samples are more closely associated with the negative carbonyl peak at 1740 cm^–1^. Additionally, films differ from powder in PC3, where the biomaterial samples are positively influenced by the C=O peak at 1740 cm^–1^, whereas the film samples show a negative correlation with the C=C peak at 1615 cm^–1^ and the ester C–O stretching peak at 1312 cm^–1^. Notably, the C=C peak for the films was more intense than that for the powder, likely due to the conjugation effect of vibrations with the carbonyl group. Furthermore, the PC3 scores revealed that the films that deviated from their peers were those with the lowest irrigation index, suggesting a potential correlation between irrigation percentage and the spectral characteristics of the films.

According to Figure 11, the powder and film samples from cactus exhibited a clear correlation with the percentage of irrigation. To validate this observation, an additional Principal Component Analysis (PCA) model was constructed using only the spectra of this cactus genotype, which revealed that the samples displayed random behavior in relation to irrigation. In the PCA model focused solely on spectra (Figure 11), the OEM variety samples demonstrated distinct behavior. As illustrated in Figure 11, there is a correlation between the samples located in the negative quadrant of the PC1 scores and the conjugation peak of C=C with C=O at 1615 cm^–1^, which corresponds to negative values in the loadings graph (Figure 11B). This observation suggests that samples developed with little or no irrigation produced films with a lower carbonyl index, as the intensity of the film’s C=C signal increased at ETc percentages of 80 and 120%. This result is also evident in the spectra graph, where this peak exhibits greater intensity under these preparation conditions. It is important to note that the findings of^11^ indicated that for ethanol-extracted mucilage, films produced during the wet and wet–dry transition seasons were superior to those produced in the dry season. This contradicts the data presented in this manuscript. However, several significant differences must be considered. First, the powder obtained through solvent-free methods possesses a greater diversity of phytochemicals, both organic and inorganic, including pigments, resulting in a pigmented material (Figure 12A,B). This led to a different protocol for producing the biopolymeric film. Second, the irrigation management applied to the plants allowed for tighter control over water availability for the cladodes, which differed from the conditions observed by.^11^ The rainy season, during which the cladodes were collected, is characterized by fluctuations in water availability; some days experience heavy rainfall, while on others, the plants endure microdrought conditions. Consequently, a strict irrigation schedule was implemented to standardize water availability for the cladodes as much as possible.

**Figure 11:**
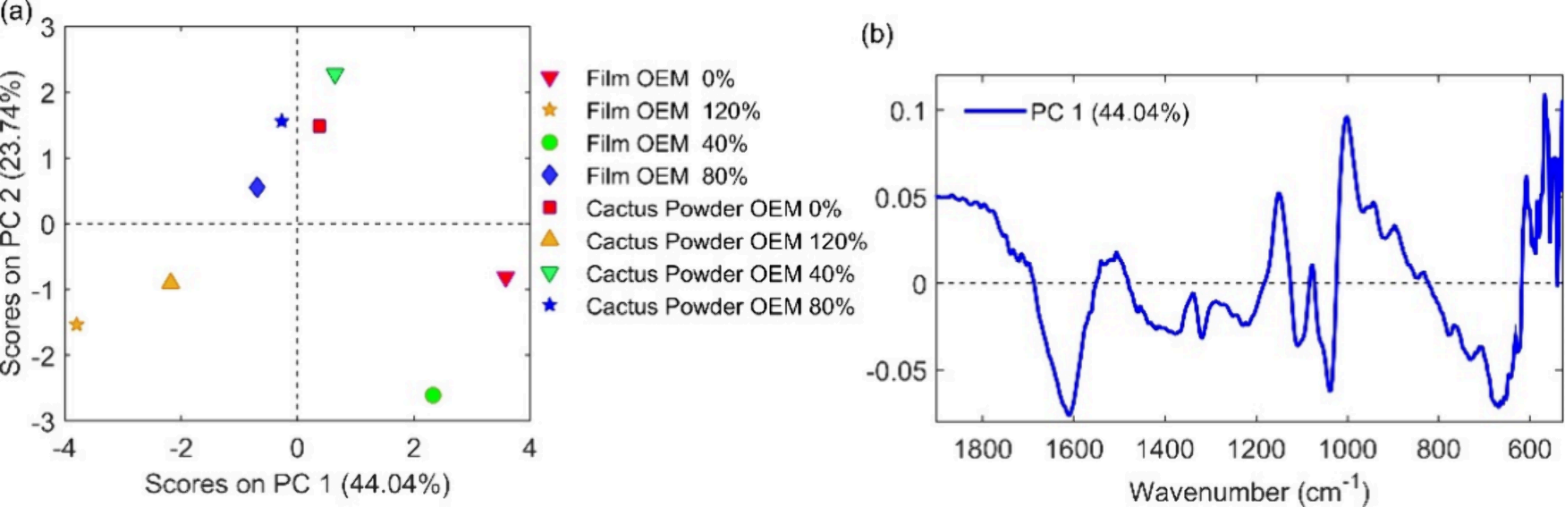
PCA biplots based on infrared spectral data of cladode powders and biopolymeric films from *Opuntia stricta* (Haw.) Haw (OEM), cultivated under different irrigation regimes, immediately after harvesting the cladodes and after treatments at 0, 40, 80, and 120% of ETc. (A) Biplot of scores for the first two principal components, showing the grouping of samples according to genotype and irrigation regime. (B) Biplot of loadings for the first two principal components, identifying the main spectral regions responsible for the variance observed among the samples.

**Figure 12:**
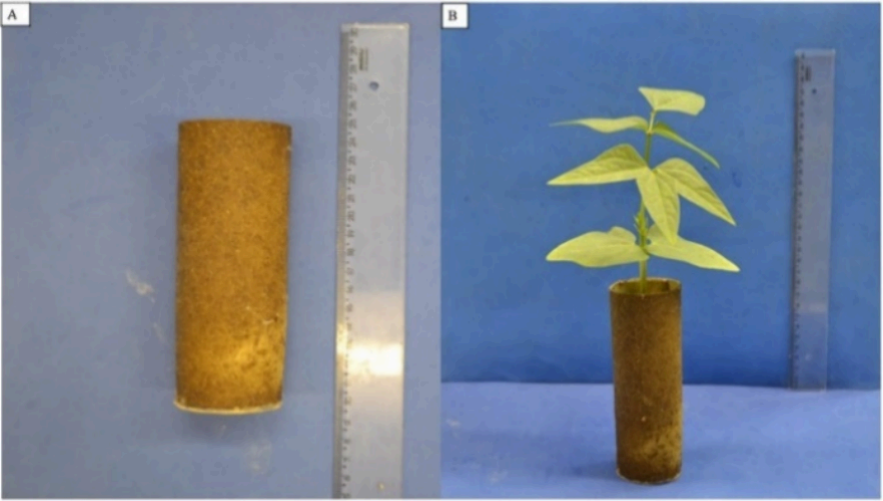
Biopolymeric structure produced from cactus: *Nopalea cochenillifera* (L.) Salm-Dyck–IPA (A) and *Opuntia stricta* (Haw.) Haw–OEM (B). Photographs taken by the authors.

Finally, the produced biomaterial demonstrated significant potential for use in packaging for vegetable seedling production, offering an autonomous and fully biodegradable packaging (Figure 12A,B).

Although it is known that biopolymer films made from cacti lack durability due to their low resistance, such films can be entirely viable when produced and utilized in seedling trays. In this context, it is suggested that cactus IPA and OEM cultivated with reduced irrigation (up to 80% of ETc), as recommended for semiarid conditions, could be ideal for the production of biopolymeric films. These films are likely to exhibit greater resistance and potentially enhanced durability compared to those produced with higher ETc values. However, this assertion requires further investigation, and additional research on this topic is essential.

## 4 Conclusions

The physicochemical, optical, mechanical, and macro- and microstructural properties of cactus dried cladode powder and biopolymeric films derived from it were analyzed. A multivariate analysis of the physicochemical data and infrared spectra was conducted. The powder from the OEM genotype exhibited higher values for yield, titratable acidity, total soluble carbohydrates, total phenolic content, and potassium. In contrast, the powder from the IPA genotype displayed lower values for pH, electrical conductivity, phosphorus, and sodium. Furthermore, as the water levels available to the plants ranged from 0 to 80% of the crop evapotranspiration (ETc), the powder from both genotypes became more acidic, with increased contents of total phenolic content, total carbohydrates, and electrical conductivity, indicating its potential for incorporation into food products.

The biopolymeric films produced from the OEM genotype exhibited superior mechanical properties, water barrier performance, microstructure, and thermal stability compared to those derived from the IPA genotype. Spectral data and multivariate analysis clearly demonstrated a correlation between water availability, as measured by the ETc irrigation regime, and the physicochemical properties of the powder and its biopolymer films from the OEM genotype. This analysis indicated that cultivating crops with lower ETc values resulted in films with reduced carbonyl content and enhanced quality. Therefore, field management practices that maintain lower ETc levels promote the production of more desirable biopolymer films for the biodegradable packaging industry, including films suitable for growing seedlings.
